# Impact of rural family physician program on child mortality rates in Iran: a time-series study

**DOI:** 10.1186/s12963-017-0138-0

**Published:** 2017-06-02

**Authors:** Shohreh Naderimagham, Hamidreza Jamshidi, Alireza Khajavi, Farhad Pishgar, Ali Ardam, Bagher Larijani, Zohreh Mahmoudi, Alireza Jeddian, Hamid Reza Bahrami-Taghanaki, Farshad Farzadfar

**Affiliations:** 1 Non-Communicable Diseases Research Center, Endocrinology and Metabolism Population Sciences Institute, Tehran University of Medical Sciences, Tehran, Iran; 2 Endocrinology and Metabolism Research Center, Endocrinology and Metabolism Clinical Sciences Institute, Tehran University of Medical Sciences, Tehran, Iran; 3 School of Medicine, Department of Pharmacology, Shahid Beheshti University of Medical Sciences, Tehran, Iran; 4 Faculty of Paramedical Sciences, Shahid Beheshti University of Medical Sciences, Tehran, Iran; 5 Liver and Pancreatobiliary Diseases Research Center, Digestive Diseases Research Institute, Tehran University of Medical Sciences, Tehran, Iran; 6 Complementary and Chinese Medicine, Persian and Complementary Medicine Faculty, Mashhad University of Medical Sciences, Mashhad, Iran

**Keywords:** Child Mortality, Family Physician, Health Care Reform, Mortality, Infant Mortality, Iran, Rural Health Services, Rural Population

## Abstract

**Background:**

The rural family physician program and social protection scheme were started in Iran about 10 years ago, and no comprehensive study has been carried out to investigate the effects of this program on mortality-related health indicators yet. The present study aims to examine the impacts of implementation of the family physician program and rural insurance program, which was launched in June 2005, on neonatal (NMR), infant (IMR), and under-5-year (U5MR) mortality rates in rural areas of Iran between 1995 and 2011, using a time-series analysis.

**Methods:**

Three segmented regression models were built to evaluate the effects of the program on NMR, IMR, and U5MR, and several independent variables were entered into the models, including annual incremental effect of the program (variable of interest), time effect, behvarz density, effect of the family physician and rural insurance programs, as well as socioeconomic variables including years of schooling, wealth index, sex ratio, and logarithmic scales of rural population size in each area. Data were gathered from secondary sources and other studies. Data pertaining to the year 2007 were excluded from the final analysis due to their inaccuracy.

**Results:**

Our results show that the incremental effect of implementing the rural family physician program is associated with significant reductions in NMR (*β* = − 0.341. *p* − *value* = 0.003) and IMR (*β* = − 0.016. *p* − *value* = 0.009). Although the association between this effect and reductions in U5MR were evident, they were not statistically significant (*β* = − 0.003. *p* − *value* = 0.542). Moreover, wealth status of inhabitants was associated with reductions in NMR (*β* = − 0.889. *p* − *value* = 0.001), IMR (*β* = − 0.052. *p* − *value* < 0.001), and U5MR (*β* = − 0.055. *p* − *value* < 0.001) in the time period of the study.

**Conclusions:**

In this nationally representative study, we showed that implementation of the second health system reform in Iran, known as the family physician program and social protection scheme for rural inhabitants, is associated with significant reductions in NMR and IMR. However, reported reductions in U5MR were not found to be statistically associated with the launch of the program.

The advantage of this study was the ability to depict a more precise picture of the outcomes of a national-level intervention.

**Electronic supplementary material:**

The online version of this article (doi:10.1186/s12963-017-0138-0) contains supplementary material, which is available to authorized users.

## Background

The World Health Organization (WHO) introduced universal health coverage (UHC) in 2005 with the idea of providing effective health services to all people, regardless of their financial status or economic barriers. UHC not only deals with bringing about services related to treatment of diseases, it also incorporates promotive, preventive, and palliative care [1]. Several factors have been mentioned as main steps toward achieving UHC in communities or countries, including basis of a strong health system, providing affordable health services by financing health facilities using a well-run scheme, and availability of necessary medicines, medical technologies, and motivated physicians and health workers to provide people with evidence-based health services [1, 2].

**Fig. 1:**
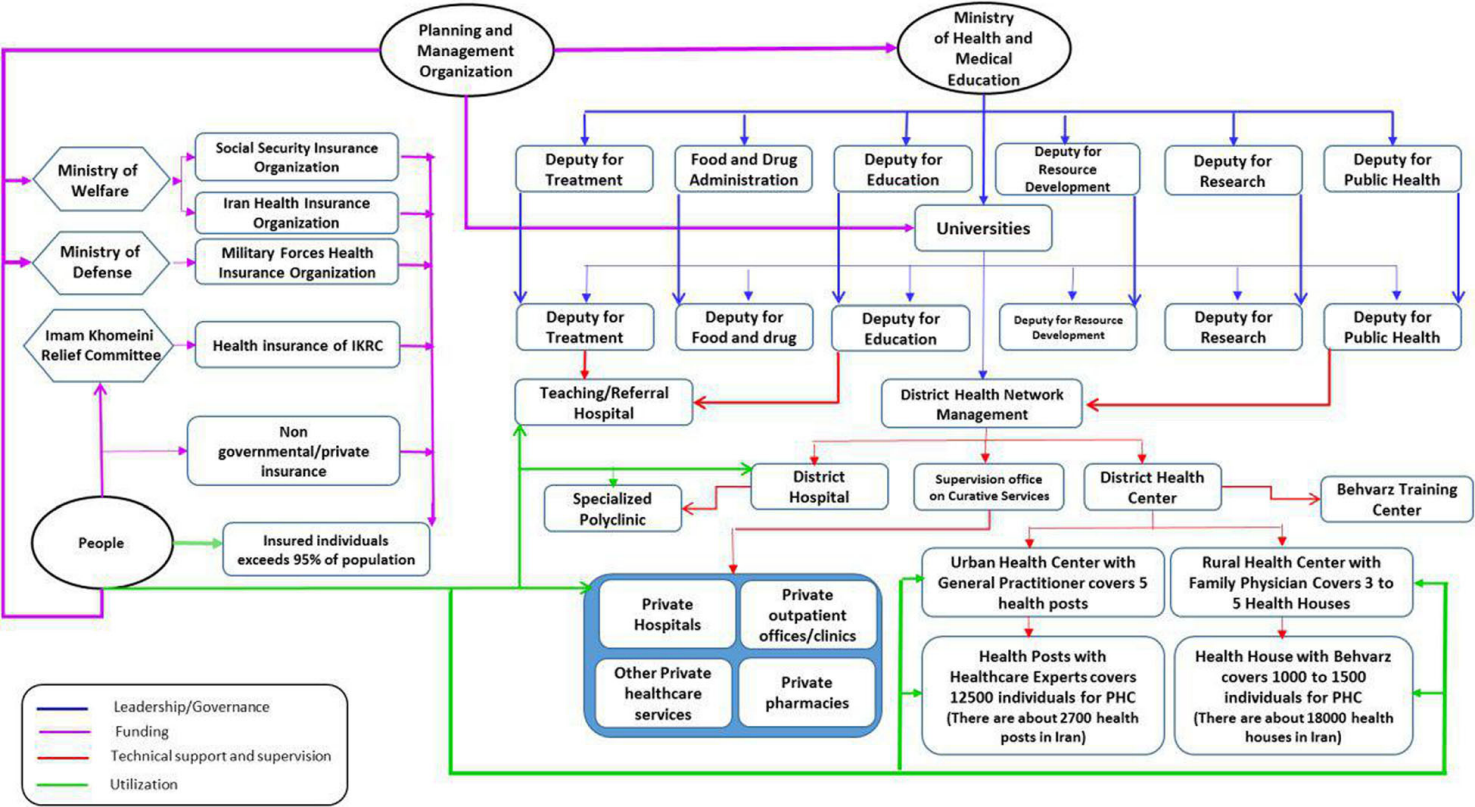
Structure and connection of different parts of the health system in Iran

The Islamic Republic of Iran has undergone two remarkable health reforms in the past three decades. The first one was the launch of the primary health care (PHC) network in the mid-1980s in rural areas of Iran, areas with less than 5,000 inhabitants in which the main occupation is farming. Health centers and health houses were launched to provide rural inhabitants with primary health services as a part of the PHC network. Providers of first-line health services, community health workers known as behvarzes in the Persian language, were selected from among local inhabitants, and health houses were staffed by one or two behvarzes who had been trained for 2 years [3, 4]. Behvarzes’ activities mostly include primary health care related to maternal and child health, vaccination, and family planning. Their activities were supervised by general physicians based in rural health centers, who regularly see patients referred by these community health workers. However, the primary health care network, budgeted by the government, did not cover access to specialists or physicians working in the private sector for rural inhabitants. The existing gap between health care utilization in urban and rural populations was the main driver for implementing another health system reform, known as the family physician program and rural health insurance plan for rural inhabitants, in June 2005. Six thousand family physicians and 4,000 midwives were employed in about 2,500 health centers in a six-month period, to provide services to rural, tribal, and urban areas with up to 20,000 residents (each province [with 2 million inhabitants on average] is made up of several districts, and each district has both rural and urban areas, so provinces are populated by both rural and urban populations). This program was an effective health sector reform with the purpose of maintaining and promoting PHC program achievements. Moreover, due to changes in payment mechanism (from “fee for service” to “per capita” and more payment for deprived areas), it facilitated access for rural inhabitants to preventive and outpatient care (with more focus on preventive services), and provided the main elements necessary for UHC in Iran’s health system, including equity in health care access, a universal service package, fairness in financial burden, and a trained health workforce [4, 5]. Figure 1 shows the current structure of the health system in Iran. As a result, following implementation of the PHC network and rural family physician program and the social protection scheme in Iran, improvements in health indicators were expected. There are several indicators, including insurance uptake and care-seeking behavior, to measure the effects of these programs, some of which had been studied before [6–8]. Among the available measures, and the one that reflects performance of the health system on a long-term basis, mortality rates are important indices and could be considered key criteria for assessing health status in countries. Neonatal (NMR), infant (IMR), and under-5 (U5MR) mortality rates are examples of mortality rate indices. It has been about 10 years since the rural family physician program and social protection scheme was started in Iran, and no comprehensive study has been carried out to investigate its effects on mortality-related health indicators. We conducted this nationally representative study to evaluate the impacts of the family physician program and social protection scheme for rural inhabitants (rural health insurance plan) on child mortality rates in the Iranian population between 1995 and 2011. Over this period Iran was not involved in any conflicts with its neighboring nations; however, several sanctions from European nations and the United States were imposed on the Iranian economy in these years. The effect of UN sanctions on Iran’s economy accelerated from 2012 until 2015 (out of the data range of our study), after the “Joint Comprehensive Plan of Action” [9].

## Methods

The goal of the present study was to investigate the impact of implementing the family physician and rural insurance program, which was launched in June 2005, on child mortality rates (NMR, IMR, and U5MR) in rural areas of Iran between 1995 and 2011, using a time-series analysis.

### Sources of data

Human resources data, including the number of behvarzes in different areas, were obtained from the data in vital horoscopes (Zij – manually administered health information system) and DTARH software (software used in surveys of primary health care systems in Iran) studies. Behvarz density was defined as average number of behvarzes per 1,000 rural inhabitants of each province. Moreover, rural area populations and death rates were collected from data of vital horoscopes.

Wealth index was calculated by the principal component analysis (PCA) method using data from a national survey of household expenditure and income by the Statistics Centre of the Islamic Republic of Iran. PCA was performed on 14 assets including the home area; number of rooms; type of materials used in home construction; type of fuel used for home heating; owning laundry machines, freezers, vacuum cleaners, personal computers, cell phones, telephones, and cars; as well as kitchens, bathrooms, and access to gas pipelines. Its values range roughly between -4 and +4. After calculating wealth at the household level, it was averaged at the provincial level, and its exact value, without any transformation or categorization, was used in the analyses. Data on years of schooling were obtained from the same study and were unified using the international standard classification of education (ISCED-1976 and -1997). Years of schooling is a discrete variable with values 0, 1,…, 30, registered for each person. No categorizations were applied to it.

Data pertaining to 2007 were excluded from the final analysis, due to their inaccuracy. In more detail, the reason was the notable inconsistencies between human resources data (behvarz density) for this year and the years before and after it (see Additional file 1: Figure S1). Due to the fact that the behvarzes are government employees for 30 years’ service, their number could not exhibit such a drop in a year. Thus, we considered that the data for this year might be invalid and they were removed from the analyses. All the data were gathered at the level of districts; however, due to some inconsistencies, we performed our analysis at the level of provinces. As a result, data for 16 years in 25 provinces (the number of provinces in 1995) yielded values of 400 observations.

### List of variables

Three response variables were recognized in the segmented regression model of this study, neonatal mortality rate (NMR), which is defined as the number of deaths among children younger than 28 days per 1,000 live births; infant mortality rate (IMR), which is the number of deaths per 1,000 live births in children younger than 1 year; and under-5 mortality rate (U5MR), defined as the number of deaths among children under 5 years per 1,000 live births. However, since the IMR values in the time frame of the study had significant deviations from the normal distribution, logarithmic-transformed values were used in the regression model as the response variables.

Several independent variables were entered into the model including annual incremental effect of the rural family physician program and social protection scheme (the main variable of interest), time effect, behvarz density, and effect of the program. Moreover, four socioeconomic variables, years of schooling, wealth index, sex ratio, and logarithmic scales of rural population size in each area, were used to increase efficiency and power of the model (Table 1).

**Table 1:** Mean levels of covariates between years of 1995–2004 and 2005–2011. Data are shown as values (standard errors)

Covariates	1995–2004	2005–2011
Wealth index	-1.855 (0.067)	-0.017 (0.069)
Years of schooling	3.562 (0.049)	5.046 (0.084)
Behvarz density	1.371 (0.017)	1.571 (0.031)
Sex ratio	1.049 (0.001)	1.044 (0.002)
Population	791,492.400 (33,320.83)	801,930.600 (45,187.72)

### Statistical analysis

The segmented regression model we used in our study is as follows:

$$ {y}_i={\beta}_0+{\beta}_1 B{D}_i+{\beta}_2 F{S}_i+{\beta}_3 AI{E}_i+{\beta}_4{T}_i+{\beta}_5 Y O{S}_i+{\beta}_6 W{I}_i+{\beta}_7 S{R}_i+{\beta}_8 L{P}_i+{\beta_9}_d{P_i}_d $$

In this model, y is defined as the response variable, BD as the behvarz density, FS as the effect of the family physician program and rural insurance program, which is an indicator of years before the program (*FS* = 0) or after establishing the program (*FS* = 1). In fact, FS retains the segmented nature of the model. AIE shows the annual incremental effect of the program, with values of 0 in years before the program, and 1 through 7 for years after the intervention. The time effect, is shown as T and is entered in a scaled form in the model changing from 1 through 17 corresponding to years. The four socioeconomic covariates, YOS as the years of schooling, WI as the wealth index, SR as the sex ratio, and LP as the logarithmic scale of the rural population size, were also entered in the model. Years of schooling was counted as a discrete variable with values 0, 1,…,30 registered for each person, and no categorizations were applied on it. Finally, P_d_ as fixed effects of provinces are put in the model to control the effect of any unmeasured confounder.

Considering the fact that child mortality could be affected by the proportion of males in the population, the sex ratio was added to control any variation in the male/female proportion of the population. It equals the number of males divided by the number of females in the population.

In the segmented regression model, the expectation is that by accumulating the years after performing the intervention, the effect of this intervention on the response variable becomes greater and greater. In fact, the annual incremental effect could be regarded as the average annual effect from the beginning of the intervention (2005) until the end year of the study (2011). Hence, the annual incremental effect takes value 0 for years before intervention, and values 1 through 7 for years 2005–2011. Also, to shed some light on the status of the responses and covariates at the subnational level, their mean values are presented in the appendix (Additional file 2: Table S1).

## Results

**Fig. 2:**
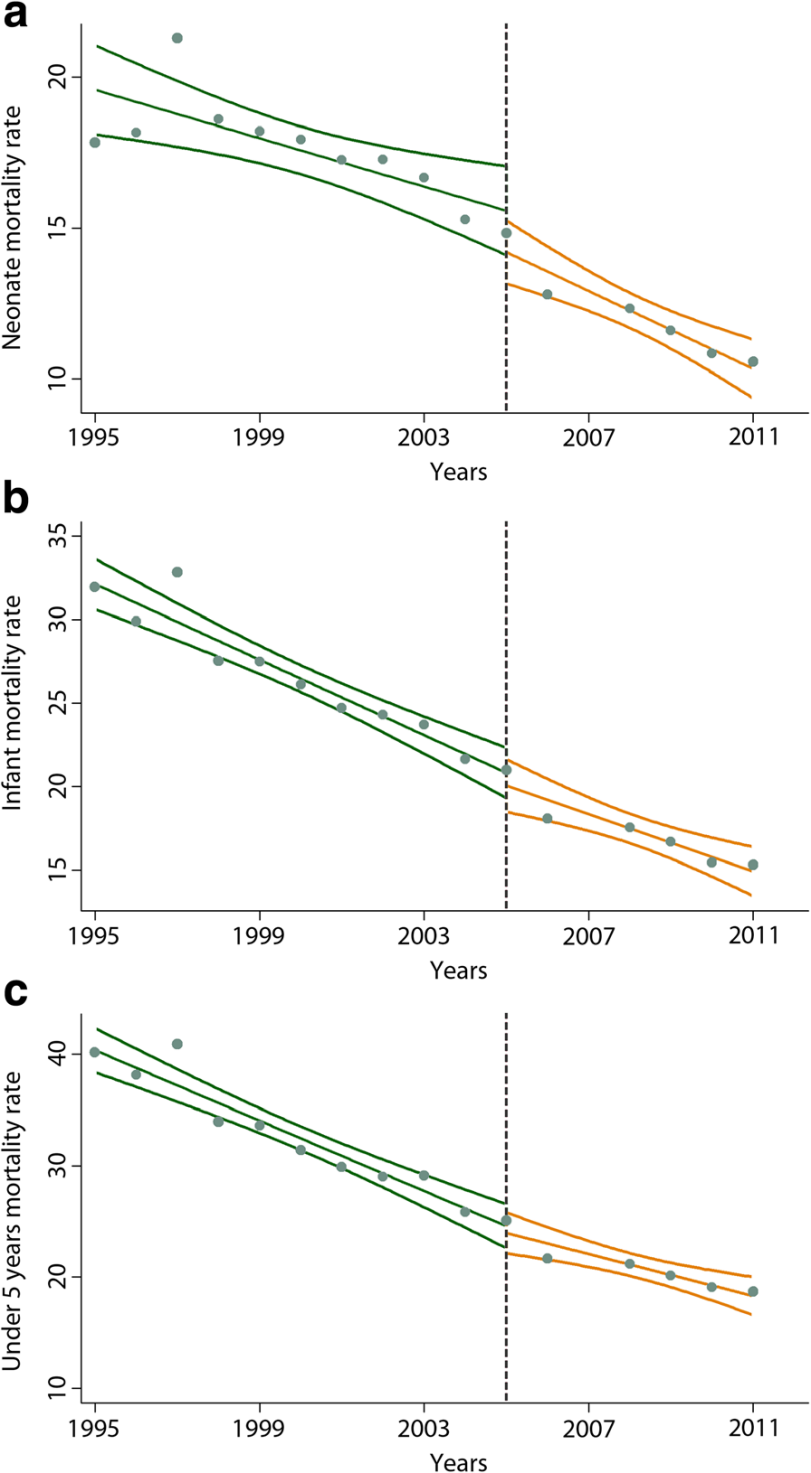
Trends in neonatal, infant, and under-5 mortality rates in rural areas of Iran, 1995–2011. Neonatal (**a**), infant (**b**), and under-5 (**c**) mortality rates in rural areas of Iran, with respect to the launch of the rural family physician and social protection program (the vertical dashed line) are shown in the graphs. A fitted line with its confidence interval is also shown in each figure to present the trend of values in the time period. Data pertaining to 2007 were excluded from the analysis due to inconsistency

Neonatal, infant, and under-5 mortality rates showed a decreasing trend between 1995 and 2011, declining from 17.84, 31.95, and 40.17 in 1995 to 10.56, 15.31, and 18.67, respectively, in 2011 (Fig. 2).

To provide an overview of Iran’s location and provincial divisions in 1995, a map has been provided in the appendix (Additional file 3: Figure S2).

### Effects of the rural family physician program and social protection scheme on neonatal mortality rate (NMR)

A segmented regression model was built to evaluate effects of the rural family physician program and the social protection scheme on NMRs between 1995 and 2011. Values of NMR in the period of the study were normally distributed and thus did not require any further transformation. In the model, NMR was recognized as the response variable, and eight variables, including effect of the program, annual incremental effect, behvarz density and time effect, as well as four other socioeconomic variables, were entered as covariates. Regression coefficients and corresponding p-values are summarized in Table 2.

**Table 2:** Regression coefficients for models of neonatal, infant, and under-5 mortality rates in rural areas of Iran, 1995–2011

Variable	Neonatal mortality rate (NMR)	Infant mortality rate (IMR)	Under-5 mortality rate (U5MR)
Behvarz density	-0.108 (-2.09 – 1.87)	-0.021 (-0.13 – 0.08)	-0.012 (-0.11 – 0.09)
Effect of rural family physician program and social protection scheme	-0.906 (-1.94 – 0.13)	0.002 (-0.05 – 0.06)	0.006 (-0.04 – 0.06)
Annual incremental effect of rural family physician program and social protection scheme	-0.341* (-0.57 – -0.11)	-0.016* (-0.03 – -0.01)	-0.003 (-0.01 – 0.01)
Time effect	-0.231* (-0.39 – -0.07)	-0.034** (-0.04 – -0.03)	-0.040** (-0.05 – -0.03)
Years of schooling	0.237 (0.35 – 0.82)	0.024 (-0.01 – 0.06)	0.020 (-0.01 – 0.05)
Wealth index	-0.889* (-1.43 – -0.35)	-0.052** (-0.08 – -0.02)	-0.055** (-0.08 – -0.03)
Sex ratio	2.051 (-33.44 – 37.54)	0.904 (-0.99 – 2.80)	1.312 (-0.49 – 3.11)
Logarithmic scale of rural population size	-0.918 (-4.05 – 2.21)	-0.223* (-0.39 – 0.06)	-0.219* (-0.38 – -0.06)

Regression coefficients are shown in values (95% confidence intervals). Superscript stars correspond to significance of calculated values: * shows *p*-values > 0.01, and ** shows *p*-values > 0.001. Data on average wealth status and years of schooling are collected from national survey of household expenditure and income by the Statistics Centre of the Islamic Republic of Iran; other data are extracted from the vital horoscopes and DTARH studies

Results of the analysis showed that although NMR decreases in subsequent years (time effect, *β*_4_ = − 0.231, *p* − *value* = 0.004), cumulative effects of implementing the rural family physician and insurance program (annual incremental effect, *β*_3_ = − 0.341, *p* − *value* = 0.003) result in significant reductions in this mortality rate. Fig. 2a shows NMRs in rural areas of Iran before the intervention, compared to similar values after that. Moreover, in our model, lower NMRs were reported among families with higher family incomes, since the calculated coefficient for the wealth index was statistically significant (*β*_6_ = − 0.889, *p* − *value* = 0.001). However, our model did not show a role for behvarz density (*β*_1_ = − 0.108, *p* − *value* = 0.914).

### Effects of the rural family physician program and social protection scheme on infant mortality rate (IMR)

Eight covariates, the same as in the NMR regression model, were entered in the segmented regression analysis and their coefficients were calculated (Table 2). Our model showed that in spite of the effect of time in subsequent years (*β*_4_ = − 0.034, *p* − *value* < 0.001), incremental effect of implementing the program (*β*_3_ = − 0.016, *p* − *value* = 0.009) (Fig. 2b), logarithmic scale of inhabitants (*β*_8_ = − 0.223, *p* − *value* = 0.009), and economic status of families (*β*_6_ = − 0.052, *p* − *value* < 0.001) had significantly reduced IMR between 1995 and 2011. However, increasing behvarz density was not shown to have decreasing effects on IMR (*β*_1_ = − 0.021, *p* − *value* = 0.687).

### Effects of the rural family physician program and social protection scheme on under-5 mortality rate (U5MR)

A logarithmic transformation was used to make U5MR values normal. The transformed values were recognized as response variables and the same eight covariates were entered in a segmented regression model (Table 2).

Our model showed that the coefficients corresponding to density of behvarzes (*β*_1_ = − 0.012, *p* − *value* = 0.815) and annual effect of the program (*β*_3_ = − 0.003, *p* − *value* = 0.542) (Fig. 2c) did not reduce the U5MR between years of 1995 and 2011. However, coefficient of wealth index (*β*_6_ = − 0.055, *p* − *value* < 0.001), time effect (*β*_4_ = − 0.040, *p* − *value* < 0.001) and logarithmic scale of rural inhabitants (*β*_8_ = − 0.219, *p* − *value* = 0.007) were statistically significant in our analysis.

## Discussion

Previous studies showed that implementing the first health system reform in Iran, the PHC network in 1985, was linked with reductions in NMR, IMR, and U5MR indices [10–12]. In this nationwide study, we showed that implementation of the second health system reform in 2005, known as the family physician program and social protection scheme for rural inhabitants, is associated with significant reductions in NMR and IMR. However, the observed reductions in U5MR were not found to be statistically associated with launch of the program.

Several papers studied relationships between health-related outcomes and density of health workforce around the world. Some of them showed that higher densities of health workforce are associated with improved health outcomes, e.g., lower mortality rates, higher vaccination coverage, or better outcomes of diabetes [13–15]. In spite of ecological, social, economic, and demographic differences, we reported lower mortality rates among particular age groups after introduction of the family physician and social protection program, a trained health workforce, in rural areas of Iran. However, our models did not support such associations between behvarz density and child mortality rates.

To our knowledge, the present study is the first comprehensive nationwide analysis of the effects of the Iranian second health system reform on death-related health measures, child mortality rates. We built one of the most powerful analysis methods, a segmented regression model, entering several covariates and adjusting for confounders to study trends of child mortality rates and to investigate the effects of the rural family physician program and social protection scheme on NMR, IMR, and U5MR over a period of 16 years.

Previous studies investigated the effects of the rural family physician program on mortality rates among the Iranian population using different methodologies and shorter time periods. Barati et al. studied implementation of the family physician program and rural insurance scheme and its effects on health indicators in rural areas of Iran. They reported that following the program since 2005, the NMR, IMR, and U5MR indices decreased compared to prior years. However, they used different statistical method from ours. Barati et al. carried out their work using a paired statistical test, not counting the increase in the level of literacy (especially in women) and changes in economic and social conditions. Moreover, they analyzed data from a shorter time period, 2001 to 2006 [6]. Given the timeframe of our work, 1995–2011, and the robust analysis we used, our study gives a more comprehensive picture of the effects of the family physician and rural insurance program on health indicators in Iran. Raeissi et al. investigated trends of several health indicators between 2001 and 2007, which overlaps with the time of the family physician and rural insurance program implementation in 2005. They showed that the program was associated with reductions in NMR, IMR, and U5MR after its implementation, as well as several other indicators. Except for the trends in U5MR, Raeissi et al.’s results are similar to ours. However, using a simple paired statistical analysis, confined to only 10 health centers and 65 health houses in a small district, Fuman (Gilan province, north of Iran) and a short time period (2001–2007) limits generalization of Raeissi et al.’s findings to other parts of Iran [8]. In another study, Raeissi et al. investigated the impact of this health system reform on maternal and child mortality indicators in rural populations of Mashhad (Razavi Khorasan province, east of Iran). Their work failed to show significant improvements in NMR, IMR, and U5MR after implementation of the family physician program and social protection scheme [7]. One possible explanation could be the confinement of their work to Mashhad rural areas (the capital of Razavi Khorasan province), and then generalizing their findings to the whole Razavi Khorasan province. In fact, the information obtained from a restricted area is not necessarily representative of the larger geographical unit.

Moreover, although not statistically significant, they showed improvements in child mortality measures. Child and maternal mortality rates are not the most inclusive measures for studying the effects of such programs. Recruiting new health workforces has, however, been recognized to increase satisfaction and improve health and cost indicators even in more developed countries [16]. It was, therefore, not surprising to expect improvements in the measures as reflected Raeissi’s work.

Both the rural family physician program and the protection scheme, as well as the Iranian primary health care network, led to significant reductions in NMR and IMR in the health system of Iran [12]. As an example, the national immunization program, which includes vaccinations for several childhood diseases, has a mean coverage of about 99% in Iran, provided by behvarzes as a part of their defined responsibilities in the PHC program [17]. On the other hand, presence of family physicians in rural areas may have improved access to professional care for women during pregnancy, birth, and postpartum and hence controlled neonatal death rates. In other words, writers of the present study believe that the reported reductions in NMR and IMR after 2005 are primarily the results of a well-run primary health care model in Iran, and secondly, the trained workforce introduced by the rural family physician program may have played a role in this regard.

More than half of under-5 deaths are due to diseases that can be prevented or treated by easy and affordable interventions [18]. Strengthening Iran’s health system to improve accessibility of such interventions for more children in rural areas might be an important step in lowering U5MR. One of the factors that influences U5MR is malnutrition. Studies showed that 45% of mortalities in children under 5 could be attributed to nutritional factors, and death rates due to common childhood diseases such as diarrhea, pneumonia, and malaria are higher in children with malnutrition [18]. Turkey is among the countries with highest reduction in mortality rates between 1990 and 2007 in the world. Besides the launch of the family physician program, several other factors have been mentioned for the observed reduction in U5MR in that country, such as immigration from rural areas to urban areas, growing per capita income in families, reduction in family size, and increased education of women [19]. In other words, mortality in children under 5 can be influenced by various socioeconomic, cultural, demographic, and genetic variables, including family income and maternal education, the management of which is outside of family physicians’ authority.

We also studied the impact of families’ average economic status (defined using the wealth index) on the health promotion and mortality rates of children. Our work showed that lower child mortality rates are seen in areas with higher average incomes in the developing society of Iran. This relationship is well-established in the literature, and several studies had reported similar results. As examples, Goza et al. showed higher IMR among populations with lower economic status, [20] and Hales et al. reported higher IMRs in countries with lower gross domestic product (GDP) per capita and societies with higher inequalities in income distribution [21]. Moreover, we showed a relatively high coefficient for wealth index in our model of NMR, which possibly stems from social factors like greater family stability, better living conditions, fewer children, and lower levels of environmental exposure among wealthier families, as well as the relationship between the role of specialized care and facilities in improving neonatal mortality in rural areas, and availability of these services to people with higher average wealth status.

On the other hand, our work had several limitations. First of all, we did not have data on causes of child deaths, so it was impossible to comment on the impact of the rural family physician and social protection program on different causes of death. Second, we gathered data at the level of districts, but inconsistencies in the data structure required us to change our data level to provinces and hence we lost some details. Third, death rates and population sizes of rural areas were obtained from DTARH and vital horoscope studies, and slight fluctuations in these data were detected and resolved by manipulating the data. Moreover, we did not have all the potential drivers and possibly confounders to add to our models, so some of the possible covariates are missing in our work. Similarly, we did not have appropriate data to track uptake and performance of the rural family physician and insurance program over years of the study. And last, our work was an observational study, and thus we only show an association between the rural family physician program and death rates in Iran, and establishing a cause-and-effect relationship requires further study.

As another limitation, it should be noted that for assessing the effect of an intervention of this kind, there may be much more sensitive measures (such as changes in care-seeking behaviors, effective coverage of interventions, reductions in out-of-pocket health expenditures, etc.) than child mortality rates. However, we were faced with the restriction of lack of reliable annual estimates on these measures, while child mortality numbers were available and also trustworthy.

Moreover, our work might have policy implications. Cost-effectiveness of child survival interventions while delivering a service package like the Iranian rural family physician program has been shown before [22]; we showed an association between this program and improved health outcomes in our work. Although we did not investigate associations between non-communicable diseases and the rural family physician and insurance program, our paper presents findings for health decision-makers, which might help in further policymaking and resource allocation. In other words, our results hint at possible benefits of running a similar urban family physician program in developing communities like Iran, and shows the potential of trained health workforces, including family physicians, in improving child mortality rates.

## Conclusions

In conclusion, we report that establishing the family physician program and social protection scheme for the rural population of Iran in 2005 is associated with significantly decreased NMR and IMR between 1995 and 2011. Moreover, U5MR decreased during this period, although this reduction was not statistically associated with implementation of the program.

## Additional files

Additional file 1: **Figure S1.** Behvarz density at the national level, reflecting the inconsistency in the data for 2007. (TIF 279 kb)

Additional file 2: **Table S1.** Mean values of mortality rates and covariates for 25 provinces of Iran, 1995–2004 and 2005–2011. Values are shown in number of deaths per 1,000 live births and are presented as number (standard error). Years of schooling takes values between 0 and 30. Sex ratio is the number of males in the population divided by the number of females. Data from 2007 were excluded from the study due to inaccuracy. Data are extracted from the vital horoscopes study. (DOCX 20 kb)

Additional file 3: **Figure S2.** The map depicting Iran provincial divisions in 1995 (The two-letter codes in the figure are Hierarchical Administrative Subdivision Codes (HASC) of Iranian provinces from which “IR.” has been eliminated in order to avoid repetition. The full names of provinces are presented in the following table). (JPG 4615 kb)

## Abbreviations

IMR: Infant mortality rRate
ISCED: International standard classification of education
NMR: Neonatal mortality rate
PCA: Principal component analysis
PHC: Primary health care
U5MR: Under-5 mortality rate
UHC: Universal health coverage
WHO: World Health Organization
