# Supplementary figures and images for: Impact of rural family physician program on child mortality rates in Iran: a time-series study
Source: Popul Health Metr. 2017 Jun 2;15:21. doi: 10.1186/s12963-017-0138-0 (PMC5455106; doi:10.1186/s12963-017-0138-0)

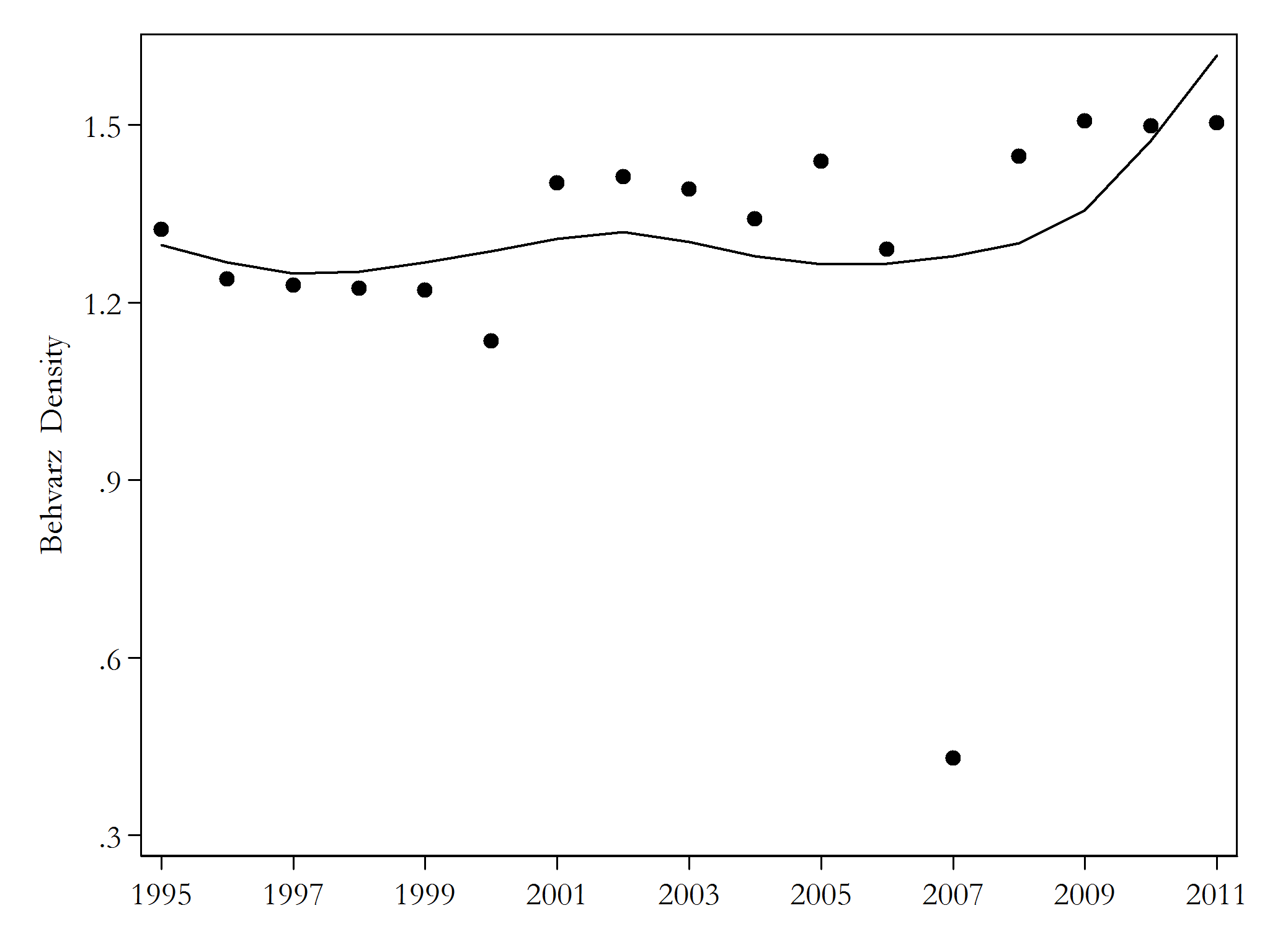

Supplement: Supplementary file 1 — Figure S1. Behvarz density at the national level, reflecting the inconsistency in the data for 2007. (TIF 279 kb) [file 12963_2017_138_MOESM1_ESM.tif]
