# Supplementary material for: Impact of rural family physician program on child mortality rates in Iran: a time-series study
Source: Popul Health Metr. 2017 Jun 2;15:21. doi: 10.1186/s12963-017-0138-0 (PMC5455106; doi:10.1186/s12963-017-0138-0)
Supplement: Supplementary file 2 — Table S1. Mean values of mortality rates and covariates for 25 provinces of Iran, 1995–2004 and 2005–2011. Values are shown in number of deaths per 1,000 live births and are presented as number (standard error). Years of schooling takes values between 0 and 30. Sex ratio is the number of males in the population divided by the number of females. Data from 2007 were excluded from the study due to inaccuracy. Data are extracted from the vital horoscopes study. (DOCX 20 kb) [file 12963_2017_138_MOESM2_ESM.docx]

**Table A.1.** **Mean values of mortality rates and covariates for 25 provinces of Iran, 1995–2004 and 2005–2011**

Values are shown in number of deaths per 1,000 live births and are presented as number (standard error). Years of schooling takes values between 0 and 30. Sex ratio is the number of males in the population divided by the number of females. Data from 2007 were excluded from the study due to inaccuracy. Data are extracted from the vital horoscopes study.

|  | Neonatal mortality rate | | Infant mortality rate | | Child mortality rate | |
| --- | --- | --- | --- | --- | --- | --- |
| Provinces | 1995–2004 | 2005–2011 | 1995–2004 | 2005–2011 | 1995–2004 | 2005–2011 |
| Markazi | 15.371 (0.859) | 9.454 (0.935) | 20.549 (1.003) | 13.659 (1.266) | 25.122 (1.249) | 17.006 (1.286) |
| Gilan | 16.705 (0.58) | 12.294 (1.002) | 21.033 (0.774) | 15.468 (1.245) | 25.241 (0.98) | 18.141 (1.542) |
| Mazandaran | 16.285 (1.312) | 10.535 (0.562) | 23.929 (2.017) | 14.827 (0.689) | 29.307 (2.495) | 17.685 (0.781) |
| Azerbaijan, East | 17.834 (1.003) | 9.239 (0.816) | 26.51 (1.697) | 14.155 (1.078) | 31.656 (2.236) | 17.231 (1.233) |
| Azerbaijan, West | 17.298 (0.412) | 12.287 (1.054) | 28.177 (1.473) | 17.038 (1.474) | 35.076 (1.96) | 20.822 (1.677) |
| Kermanshah | 21.357 (0.805) | 14.643 (0.342) | 29.461 (1.225) | 19.38 (0.554) | 35.63 (1.787) | 22.838 (0.266) |
| Khuzestan | 16.671 (0.389) | 13.622 (0.733) | 27.543 (1.036) | 19.346 (0.976) | 34.854 (1.503) | 24.179 (1.303) |
| Fars | 16.913 (0.7) | 11.013 (0.783) | 24.05 (0.996) | 15.546 (0.994) | 30.113 (1.4) | 19.11 (1.15) |
| Kerman | 15.707 (0.534) | 11.523 (0.233) | 26.381 (0.885) | 16.736 (0.494) | 36.006 (2.7) | 21.475 (0.602) |
| Khorasan, Razavi | 19.132 (0.692) | 14.129 (0.918) | 33.252 (1.945) | 20.931 (1.372) | 40.118 (2.772) | 24.651 (1.609) |
| Isfahan | 17.473 (0.76) | 11.061 (0.757) | 23.482 (0.848) | 15.869 (0.865) | 27.478 (0.966) | 18.916 (1.015) |
| Sistan & Baluchistan | 20.011 (0.61) | 14.325 (0.785) | 39.502 (1.74) | 24.271 (1.358) | 51.047 (2.618) | 30.316 (1.537) |
| Kordestan | 24.566 (1.036) | 15.42 (1.688) | 35.314 (1.884) | 20.024 (1.929) | 41.979 (2.619) | 23.327 (1.988) |
| Hamadan | 19.15 (0.997) | 12.656 (0.887) | 27.777 (1.653) | 17.243 (1.204) | 33.131 (2.04) | 20.39 (1.087) |
| Chahar Mahal & Bakhtiari | 18.005 (0.437) | 12.026 (0.813) | 25.956 (0.996) | 16.4 (0.867) | 31.562 (1.576) | 19.637 (0.959) |
| Lorestan | 20.329 (0.644) | 12.346 (1.305) | 28.802 (0.682) | 17.095 (1.528) | 34.876 (1.011) | 20.432 (1.922) |
| Ilam | 16.56 (1.068) | 13.712 (1.441) | 23.664 (1.284) | 18.325 (1.9) | 30.015 (1.945) | 21.718 (2.099) |
| Kohgiluyeh & Boyer-Ahmad | 19.27 (0.783) | 11.183 (0.618) | 30.073 (1.669) | 16.466 (1.063) | 38.654 (2.28) | 21.03 (1.401) |
| Bushehr | 20.118 (1.004) | 13.781 (1.377) | 26.901 (1.241) | 18.333 (1.463) | 32.343 (1.765) | 21.645 (1.407) |
| Zanjan | 15.675 (0.523) | 11.694 (1.019) | 23.859 (1.684) | 15.933 (1.476) | 28.458 (2.173) | 18.549 (1.51) |
| Semnan | 16.83 (0.68) | 14.899 (1.271) | 25.394 (1.479) | 21.612 (1.853) | 29.963 (1.961) | 25.351 (1.427) |
| Yazd | 16.078 (0.759) | 12.923 (0.879) | 23.246 (0.629) | 19.425 (1.427) | 29.119 (0.964) | 22.735 (1.261) |
| Hormozgan | 18.397 (0.358) | 13.925 (0.552) | 29.097 (1.275) | 19.593 (0.59) | 36.104 (1.748) | 23.908 (0.645) |
| Tehran | 14.364 (0.614) | 9.214 (0.678) | 20.78 (1.098) | 13.431 (0.892) | 25.766 (1.577) | 16.701 (1.016) |
| Ardabil | 20.802 (1.106) | 11.967 (1.273) | 30.488 (2.172) | 17.841 (1.878) | 37.902 (3.089) | 21.472 (1.926) |

Table A.1. (Continued)

|  | Wealth index | | Years of schooling | | Behvarz density | |
| --- | --- | --- | --- | --- | --- | --- |
| Provinces | 1995–2004 | 2005–2011 | 1995–2004 | 2005–2011 | 1995–2004 | 2005–2011 |
| Markazi | -1.691 (0.144) | 0.231 (0.241) | 3.628 (0.119) | 4.721 (0.4) | 1.367 (0.065) | 1.713 (0.128) |
| Gilan | -1.952 (0.168) | 0.101 (0.236) | 4.244 (0.108) | 5.628 (0.364) | 1.222 (0.064) | 1.652 (0.064) |
| Mazandaran | -0.979 (0.203) | 0.6 (0.149) | 4.316 (0.135) | 5.518 (0.337) | 1.333 (0.069) | 1.525 (0.029) |
| Azerbaijan, East | -2.265 (0.163) | 0.096 (0.214) | 3.227 (0.141) | 4.733 (0.388) | 1.478 (0.019) | 1.544 (0.022) |
| Azerbaijan, West | -2.318 (0.214) | -0.247 (0.143) | 2.629 (0.101) | 4.17 (0.406) | 1.352 (0.014) | 1.412 (0.02) |
| Kermanshah | -2.447 (0.148) | -0.613 (0.217) | 3.464 (0.129) | 4.992 (0.348) | 1.473 (0.048) | 1.728 (0.017) |
| Khuzestan | -1.169 (0.201) | 0.302 (0.205) | 3.232 (0.14) | 4.944 (0.42) | 1.155 (0.028) | 1.192 (0.074) |
| Fars | -1.204 (0.185) | 0.423 (0.155) | 4.078 (0.185) | 5.634 (0.356) | 1.011 (0.027) | 0.899 (0.161) |
| Kerman | -1.822 (0.147) | -0.76 (0.205) | 3.865 (0.138) | 5.126 (0.369) | 1.589 (0.128) | 1.511 (0.043) |
| Khorasan, Razavi | -2.093 (0.175) | -0.702 (0.19) | 3.272 (0.137) | 4.37 (0.36) | 1.15 (0.025) | 1.233 (0.049) |
| Isfahan | -0.491 (0.204) | 1.293 (0.087) | 4.466 (0.162) | 5.736 (0.279) | 1.391 (0.103) | 2.092 (0.123) |
| Sistan & Baluchistan | -4.1 (0.239) | -1.68 (0.247) | 2.105 (0.122) | 3.675 (0.422) | 1.576 (0.055) | 1.471 (0.046) |
| Kordestan | -2.689 (0.2) | -0.786 (0.197) | 2.589 (0.138) | 4.048 (0.373) | 1.456 (0.057) | 1.582 (0.016) |
| Hamadan | -1.96 (0.146) | 0.044 (0.22) | 3.438 (0.145) | 4.814 (0.337) | 1.152 (0.027) | 1.329 (0.013) |
| Chahar Mahal & Bakhtiari | -1.991 (0.166) | 0.086 (0.288) | 3.813 (0.171) | 5.388 (0.464) | 1.257 (0.069) | 1.198 (0.067) |
| Lorestan | -2.706 (0.173) | -0.733 (0.312) | 3.571 (0.181) | 5.196 (0.409) | 1.233 (0.041) | 1.469 (0.028) |
| Ilam | -1.988 (0.215) | 0.034 (0.204) | 3.871 (0.221) | 5.767 (0.458) | 1.521 (0.034) | 1.662 (0.122) |
| Kohgiluyeh & Boyer-Ahmad | -3.277 (0.219) | -0.445 (0.335) | 3.647 (0.208) | 5.55 (0.367) | 1.552 (0.016) | 1.816 (0.09) |
| Bushehr | -0.881 (0.23) | 1.002 (0.07) | 4.207 (0.204) | 6.066 (0.284) | 1.372 (0.083) | 1.521 (0.019) |
| Zanjan | -2.465 (0.3) | -0.447 (0.274) | 2.769 (0.2) | 4.557 (0.408) | 1.153 (0.057) | 1.583 (0.103) |
| Semnan | -0.797 (0.184) | 0.818 (0.111) | 4.09 (0.108) | 5.316 (0.363) | 1.656 (0.069) | 2.07 (0.055) |
| Yazd | -0.467 (0.192) | 0.968 (0.102) | 4.222 (0.178) | 5.52 (0.358) | 1.909 (0.107) | 2.579 (0.131) |
| Hormozgan | -1.56 (0.193) | -0.583 (0.165) | 3.127 (0.159) | 4.867 (0.361) | 1.196 (0.04) | 1.37 (0.051) |
| Tehran | -0.578 (0.175) | 0.922 (0.106) | 4.209 (0.095) | 5.27 (0.312) | 1.102 (0.032) | 1.33 (0.06) |
| Ardabil | -2.478 (0.224) | -0.357 (0.232) | 2.98 (0.124) | 4.538 (0.366) | 1.615 (0.047) | 1.796 (0.026) |

Table A.1. (Continued)

|  | Sex ratio | | Population | |
| --- | --- | --- | --- | --- |
| Provinces | 1995–2004 | 2005–2011 | 1995–2004 | 2005–2011 |
| Markazi | 1.022 (0.004) | 1.03 (0.002) | 496,176.6 (22,266.4) | 433,959.3 (38,911.29) |
| Gilan | 1.024 (0.002) | 1.022 (0.001) | 1,257,168 (28,228.81) | 1,051,444 (36,830.29) |
| Mazandaran | 1.009 (0.001) | 1.016 (0.002) | 2,082,172 (54,082.12) | 2,196,842 (24,046.46) |
| Azerbaijan, East | 1.05 (0.001) | 1.052 (0.001) | 1,121,246 (15,757.56) | 1,132,274 (19,019.36) |
| Azerbaijan, West | 1.058 (0.002) | 1.047 (0.001) | 1,063,463 (11,719.38) | 1,087,323 (17,527.52) |
| Kermanshah | 1.07 (0.001) | 1.057 (0.002) | 611,901.1 (7,857.48) | 590,787.3 (6,368.472) |
| Khuzestan | 1.051 (0.001) | 1.04 (0.001) | 1,111,493 (22,095.18) | 1,245,225 (92,541.88) |
| Fars | 1.053 (0.001) | 1.047 (0.002) | 1,575,417 (24,764.62) | 1,646,577 (85,796.8) |
| Kerman | 1.035 (0.001) | 1.034 (0.001) | 778,527.5 (46,435.14) | 875,184.7 (24,421.34) |
| Khorasan, Razavi | 1.023 (0.001) | 1.026 (0.001) | 2,126,437 (43,900.54) | 2,197,034 (79,256.51) |
| Isfahan | 1.061 (0.001) | 1.061 (0.001) | 920,496.4 (58,945.01) | 682,617 (44,942.59) |
| Sistan & Baluchistan | 1.02 (0.001) | 1.018 (0.005) | 659,435.5 (37,119.75) | 966,729.7 (31,550.03) |
| Kordestan | 1.078 (0.002) | 1.068 (0.001) | 607,257.4 (15,551.33) | 606,431.2 (5,970.073) |
| Hamadan | 1.081 (0.002) | 1.067 (0.001) | 809,480.5 (17,470.47) | 727,409.3 (6,690.072) |
| Chahar Mahal & Bakhtiari | 1.067 (0.001) | 1.065 (0.001) | 412,724.5 (16,131.83) | 448,888.3 (30,463.28) |
| Lorestan | 1.083 (0.002) | 1.068 (0.001) | 620,791.9 (12,745.18) | 660,880.7 (3,339.477) |
| Ilam | 1.076 (0.002) | 1.062 (0.011) | 206,771.5 (1,090.263) | 221,872.8 (18,190.25) |
| Kohgiluyeh & Boyer- Ahmad | 1.074 (0.003) | 1.056 (0.003) | 347,500.3 (4,137.63) | 332,341.8 (16,906.47) |
| Bushehr | 1.021 (0.002) | 1.011 (0.001) | 311,295.7 (15,738.63) | 282,689.3 (3,817.581) |
| Zanjan | 1.035 (0.004) | 1.033 (0.002) | 402,700.7 (18,620.54) | 419,812 (21,867.7) |
| Semnan | 1.029 (0.001) | 1.023 (0.001) | 143,695.2 (4,180.598) | 122,023.2 (2,545.558) |
| Yazd | 1.055 (0.002) | 1.05 (0.001) | 226,657.5 (11,972.93) | 164,549.2 (8,370.607) |
| Hormozgan | 1.034 (0.001) | 1.033 (0.003) | 540,057.6 (23,567.86) | 661,989.7 (17,915.58) |
| Tehran | 1.05 (0.002) | 1.053 (0.002) | 870,892.3 (29,195.31) | 817,395.2 (35,690.54) |
| Ardabil | 1.064 (0.002) | 1.057 (0.001) | 483,552.4 (14,841.96) | 475,986.5 (6,222.281) |
